# High‐throughput screening of argan oil composition and authenticity using benchtop ^1^H NMR

**DOI:** 10.1002/mrc.5023

**Published:** 2020-04-06

**Authors:** Yvonne Gunning, Alex J. Jackson, Joshua Colmer, Fouad Taous, Mark Philo, Rachel M. Brignall, Tibari El Ghali, Marianne Defernez, E. Kate Kemsley

**Affiliations:** ^1^ Core Science Resources Quadram Institute Bioscience Norwich UK; ^2^ Anthony Hall Group Earlham Institute Norwich UK; ^3^ Structural and Isotopic Analysis Laboratory Centre National de l'Energie des Sciences et des Techniques Nucléaires (CNESTEN) Rabat Morocco; ^4^ Oxford Instruments Magnetic Resonance Oxford Instruments Oxford UK

**Keywords:** argan oil, authenticity, benchtop NMR spectroscopy, edible oil, proton NMR, triglyceride, unsaturates

## Abstract

We use 60‐MHz benchtop nuclear magnetic resonance (NMR) to acquire ^1^H spectra from argan oils of assured origin. We show that the low‐field NMR spectrum of neat oil contains sufficient information to make estimates of compositional parameters and to inform on the presence of minor compounds. A screening method for quality and authenticity is presented based on nearest‐neighbour outlier detection. A variety of oil types are used to challenge the method. In a survey of retail‐purchased oils, several instances of fraud were found.

## Graphical abstract

We use 60‐MHz benchtop nuclear magnetic resonance (NMR) to acquire ^1^H spectra from argan oils of assured origin. We show that the low‐field NMR spectrum of neat oil contains information sufficient for obtaining composition values and informing on the presence of minor compounds. A screening method for quality and authenticity is presented based on nearest‐neighbour outlier detection. A variety of oil types are used to challenge the method. In a survey of retail‐purchased oils, several instances of fraud were found.

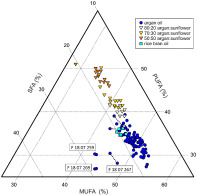

## 1 INTRODUCTION

Native to Morocco, the argan tree (*Argania spinosa* L.) is a slow‐growing tree widely cultivated for its crop of small, round, fleshy fruits. Traditionally, argan oil was produced by grinding the kernels by hand, although today, modern mechanical presses are often used. It is recognised as a Protected Geographical Indication product of Morocco and registered in the European ‘DOOR’ database (Database of Origin and Registration, Dossier Number: MA/PGI/0005/00906, 2011).

In recent years, argan oil has become one of the most expensive edible oils in the world due to its reputed pharmacological properties.^1^ In addition to culinary usage, it is also used as an ingredient in cosmetics, and this has significantly increased its value as an export commodity. The rising demand for argan oil has led to a growing number of cases of economically motivated adulteration. One of the most common adulterants is thought to be sunflower oil because of its relatively low cost and ready availability.

A typical nut oil, argan oil is composed largely of mixed triglyceride esters (TAGs) of monounsaturated (MUFA), polyunsaturated (PUFA) and saturated fatty acids (SFAs). It also contains minor components such as free fatty acids, polyphenols, tocopherols, sterols, squalene and triterpene alcohols. Various analytical techniques have been used to investigate the composition of argan oil, including high‐performance liquid chromatography,^2^ liquid chromatography–mass spectrometry^3^ and high‐field NMR spectroscopy.^4^ However, these laboratory‐based technologies are unlikely to see deployment into the sector. There remains a need for simple, robust and low‐cost analytical methods that can assure quality and detect adulteration of argan oil at commercially significant levels.

Benchtop NMR spectroscopy has the potential to meet these needs. Built using permanent magnets, these spectrometers are robust, require no services apart from electricity and can be installed in locations other than research laboratories. Typically operating at field strengths of 1.4 T (60 MHz), they have much lower capital and running costs than entry‐level high‐field (7.1 T, 300 MHz) instruments. Several recent studies have assessed the feasibility of using benchtop ^1^H NMR to authenticate edible oils, including olive,^5^ perilla^6^ and various common vegetable oils.^7^ However, none have examined argan oils.

In the present work, we use 60‐MHz benchtop nuclear magnetic resonance (NMR) to acquire ^1^H spectra from a collection of argan oils of assured origin. We show that the low‐field NMR spectrum of neat oils contains sufficient information for making useful estimates of MUFAs, PUFAs and SFAs and can also inform on the presence of more minor compounds. A screening method for quality and authenticity is presented based on nearest‐neighbour outlier detection, and a variety of oil types are used to challenge the method. Using this approach, we conduct a survey of retail‐purchased oils bearing the labelling claim ‘100% argan oil’; several instances of likely fraud were identified.

## 2 MATERIALS AND METHODS

### 2.1 Samples

#### 2.1.1 Authentic argan oils (designated as ‘A’ samples)

47 argan oils of known provenance were provided by Centre National de l'Energie des Sciences et des Techniques Nucléaires (CNESTEN; Morocco). These samples were collected as part of a Food and Agriculture Organization of the United Nations and International Atomic Energy Agency (FAO/IAEA) Coordinated Research Project ‘Field‐deployable Analytical Methods to Assess the Authenticity, Safety and Quality of Food’ (project code D52040). Details of the origin, age and storage history of these samples are given in Table 1.

**TABLE 1:** Details of the authentic argan oil samples

Sample ID	Geographic origin			Year of fruit sampling	Month/year of oil production	Extraction method	Storage
	Region	Latitude	Longitude				
F 18 07 222	Taroudante	30.754	−8.515	2016	Mar‐18	Modern mechanical	Stainless steel barrel
F 18 07 223	Taroudante	30.379	−8.694	2017	Feb‐18	Modern mechanical	Stainless steel barrel
F 18 07 224	Taroudante	30.304	−8.487	2017	Not recorded	Modern mechanical	Stainless steel barrel
F 18 07 225	Taroudante	30.304	−8.487	2017	Sep‐18	Modern mechanical	Stainless steel barrel
F 18 07 226	Taroudante	30.379	−8.694	2017	Apr‐18	Modern mechanical	Stainless steel barrel
F 18 07 227	Taroudante	30.506	−8.609	2017	May‐18	Modern mechanical	Stainless steel barrel
F 18 07 228	Taroudante	30.609	−9.077	2017	Apr‐18	Modern mechanical	Stainless steel barrel
F 18 07 229	Essaouira	31.282	−9.719	2017	Apr‐18	Modern mechanical	Stainless steel barrel
F 18 07 230	Taroudante	30.605	−8.778	2017	May‐18	Modern mechanical	Stainless steel barrel
F 18 07 231	Taroudante	30.705	−8.886	2017	Not recorded	Modern mechanical	Stainless steel barrel
F 18 07 232	Tiznite	29.631	−9.389	2017	Feb‐18	Modern mechanical	Stainless steel barrel
F 18 07 233	Chtouka ait baha	30.075	−9.046	2014	Apr‐16	Modern mechanical	Stainless steel barrel
F 18 07 234	Tiznite	29.463	−9.664	2017	May‐18	Modern mechanical	Stainless steel barrel
F 18 07 235	Sidi Ifni	29.336	−9.642	2017	Apr‐18	Modern mechanical	Stainless steel barrel
F 18 07 236	Sidi Ifni	29.361	−9.732	2017	Jan‐18	Modern mechanical	Stainless steel barrel
F 18 07 237	Tiznite	29.601	−9.906	2017	Apr‐18	Modern mechanical	Stainless & plastic
F 18 07 238	Tiznite	29.631	−9.389	2017	Mar‐18	Modern mechanical	Stainless steel barrel
F 18 07 239	Chtouka ait baha	30.075	−9.046	2017	Not recorded	Modern mechanical	Stainless steel barrel
F 18 07 240	Tiznite	29.463	−9.664	2017	May‐18	Modern mechanical	Stainless steel barrel
F 18 07 241	Sidi Ifni	29.361	−9.732	2017	Apr‐18	Modern mechanical	Stainless steel barrel
F 18 07 242	Tiznite	29.601	−9.906	2017	May‐18	Modern mechanical	Stainless steel barrel
F 18 07 243	Agadir Idaou Tanan	30.528	−9.333	2017	Apr‐18	Modern mechanical	Stainless steel barrel
F 18 07 244	Chtouka ait baha	30.027	−9.235	2017	May‐18	Modern mechanical	Stainless steel barrel
F 18 07 245	Not recorded	Not recorded	Not recorded	2017	Not recorded	Modern mechanical	Stainless steel barrel
F 18 07 246	Essaouira	31.282	−9.719	2017	Jan‐18	Modern mechanical	Stainless steel barrel
F 18 07 247	Essaouira	31.282	−9.719	Not recorded	Oct‐17	Modern mechanical	Stainless steel barrel
F 18 07 248	Essaouira	31.002	−9.683	Not recorded	May‐18	Modern mechanical	Stainless steel barrel
F 18 07 249	Essaouira	31.225	−9.653	Not recorded	Mar‐18	Modern mechanical	Stainless steel barrel
F 18 07 250	Essaouira	Not recorded	Not recorded	Not recorded	Apr‐18	Modern mechanical	Stainless steel barrel
F 18 07 251	Essaouira	Not recorded	Not recorded	Not recorded	Apr‐18	Modern mechanical	Stainless steel barrel
F 18 07 252	Essaouira	31.554	−9.302	2017	May‐18	Modern mechanical	Stainless steel barrel
F 18 07 253	Chtouka ait baha	30.011	−9.412	2017	May‐18	Traditional	Stainless steel barrel
F 18 07 254	Chtouka ait baha	30.027	−9.235	2017	May‐18	Modern mechanical	Stainless steel barrel
F 18 07 255	Chtouka ait baha	30.093	−9.157	2017	May‐18	Traditional	Stainless steel barrel
F 18 07 256	Essaouira	31.306	−9.717	Not recorded	2012	Not recorded	Not recorded
F 18 07 257	Essaouira	31.444	−9.679	Not recorded	2012	Not recorded	Not recorded
F 18 07 258	Taroudante	30.5	−8.609	Not recorded	2012	Not recorded	Not recorded
F 18 07 259	Essaouira	31.549	−9.347	Not recorded	2012	Not recorded	Not recorded
F 18 07 260	Essaouira	31.214	−9.548	Not recorded	2012	Not recorded	Not recorded
F 18 07 261	Agadir Idaou Tanan	30.998	−9.682	Not recorded	2012	Not recorded	Not recorded
F 18 07 262	Chtouka ait baha	30.07	−9.155	Not recorded	2012	Not recorded	Not recorded
F 18 07 263	Chtouka ait baha	30.847	−9.737	Not recorded	2012	Not recorded	Not recorded
F 18 07 264	Taroudante	30.399	−8.688	Not recorded	2012	Not recorded	Not recorded
F 18 07 265	Sidi Ifni	Not recorded	Not recorded	Not recorded	2012	Not recorded	Not recorded
F 18 07 267	Not recorded	Not recorded	Not recorded	Not recorded	2012	Not recorded	Not recorded
F 18 07 268	Ait Baha Tafraout	29.906	−9.406	Not recorded	2013	Not recorded	Not recorded
F 18 07 269	Essouira	31.534	−9.547	Not recorded	2013	Not recorded	Not recorded

#### 2.1.2 Argan and sunflower oil mixtures (designated as ‘Mnn’ samples)

Also supplied by CNESTEN were 36 mixtures, prepared from various of the authentic argan oils with sunflower oil in three different proportions of argan:sunflower by weight: 50:50 (13 samples, ‘M50’), 70:30 (13 samples, ‘M30’) and 80:20 (10 samples, ‘M20’).

#### 2.1.3 Assorted vegetable oils (designated as ‘V’ samples)

43 assorted vegetable oils were purchased from local retailers, as follows: avocado (three), corn (two), grapeseed (two), groundnut (three), hazelnut (one), hemp (two), olive (six), rapeseed (seven), rice bran (two), sesame (two), soya (one), sweet almond (one), sunflower (five), ‘vegetable’ (blends, four) and walnut (two).

#### 2.1.4 Surveillance samples (designated as ‘C’ samples)

Twenty eight oils were purchased from various (mostly online) retailers, all of which bore the labelling claim ‘100% pure argan oil’ or equivalent.

### 2.2 Data acquisition

^1^H NMR spectra were collected from all samples using a ‘Pulsar’ benchtop NMR spectrometer (Oxford Instruments, Abingdon, UK) operating at a frequency of 60 MHz. For each acquisition, 0.6 ml of sample was pipetted into a standard disposable 5‐mm NMR tube, with no other preparation step. The 90° pulse length was 13.9 μs as determined by the machine's internal calibration cycle. For each spectrum, 32 free induction decays Free Induction Decays (FIDs) were acquired, recording 32,768 data points over a 5,000‐Hz window (dwell time = 0.2 ms) with a relaxation delay of 2 seconds. This resulted in a total recording time of less than 5 min, in line with our requirement for a high‐throughput analysis. The linewidth was maintained between 0.6 and 0.9 Hz, by daily measurement of the chloroform FWHM in a sealed standard and shimming when necessary.

A, Mnn and C samples were analysed in duplicate. The acquisitions (referred to as ‘Run 1′ and ‘Run 2′) were separated chronologically by approximately one month. V samples were collected in triplicate with typically a few days between acquisitions.

### 2.3 Data analysis

Following acquisition, FIDs were processed in MNova (Mestrelab Research, Santiago de Compostela, Spain) using an automated script file. Spectra were phase‐corrected for visual inspection, baseline correction and peak integration.

All statistical analyses were carried out using MATLAB (The Mathworks Inc, Cambridge, UK). First, calibrations for the MUFA and PUFA contents of edible oils were obtained using the V collection of spectra, through multiple linear regression of reference compositional data onto selected peak integrals. The reference values for the samples were obtained by gas chromatography–flame ionization detector (GC‐FID) of fatty acid methyl esters (FAME ; the analysis was performed by an external accredited laboratory). The calibrations were subsequently applied to the other spectral collections.

Second, a method was developed to model the authentic argan oil class. The in‐house written script uses a nearest‐neighbour type classifier to accept an incoming spectrum as a member of the class provided it is sufficiently near to another item in the class as calculated by an ensemble of distance metrics. The threshold distance is estimated empirically to be consistent with a Type 1 error rate (incorrect rejection of an authentic class member) at the desired level. Pseudocode for the algorithm is given in the Supporting Information.

## 3 RESULTS AND DISCUSSION

**FIGURE 1:**
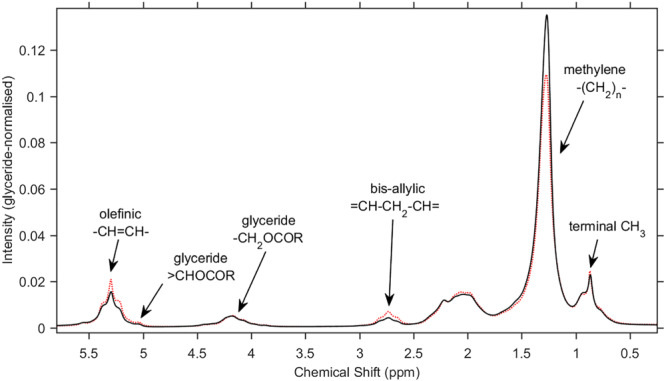
60‐MHz ^1^H spectrum of neat argan (solid black line) and sunflower (dotted red line) oils

A 60 MHz ^1^H NMR spectrum of neat argan oil is shown in Figure 1 (solid black line). A typical vegetable oil, argan oil is composed mainly (~96% by weight) of mixed TAGs of fatty acids. High‐field (300 MHz upwards) ^1^H NMR has been used extensively to examine edible oils, and comprehensive peak assignments are available.^8^ In comparison, peaks in the 60‐MHz spectrum are wider and more overlapped; complete assignment of individual resonances is not possible. However, groups of peaks can be attributed to certain proton environments, as indicated in the figure.

The spectral intensity has been scaled so that the integrated area of the glyceride bands at 4.0–4.4 ppm equals unity. Arising from protons attached to Positions 1 and 3 of the glycerol backbone, present in all TAGs irrespective of their fatty acid composition, these signals provide an obvious normalisation constant. The peaks were also used for referencing the chemical shift scale, by setting the peak maximum to 4.18 ppm in all spectra.^5^

Also shown in Figure 1 is a spectrum of neat sunflower oil (dotted red line), likewise normalised. Differences between the two oils are evident, and these are consistent with compositional information on the two oil types. Argan oil typically contains ~44%w/w MUFAs, almost all as oleic acid (C18:1); ~33%w/w PUFAs, almost all as linoleic acid (C18:2); and ~18% w/w SFAs, the majority as palmitic acid (C16:0). Sunflower oil composition varies substantially with cultivar and processing: ‘standard’ sunflower oil typically contains 20%w/w MUFAs (mostly oleic), 65%w/w PUFAs (mostly linoleic) and 10% SFAs (palmitic and stearic, C18:0). The greater PUFA content of sunflower oil is directly responsible for the evidently larger bis‐allylic peaks, and the greater total unsaturation (PUFAs + MUFAs) is responsible for the larger olefinic peaks and smaller methylene peak.

Estimating the composition of edible oils is documented for high‐field NMR spectra^9^ in which combinations of selected band integrals are used to quantify several of the most abundant unsaturated fatty acids, with a precision and accuracy comparable to that of GC methods.

At 60 MHz, the greater band overlap means that quantitation of individual fatty acids is not possible; neither is the ‘first principles’ approach, because the required peaks are insufficiently isolated for their integrals to be accurately measured. The olefinic bands, for instance, are partially overlapped by some of the glyceride resonances. However, using reference compositional data and a suitable regression method, the integrals of selected regions can yield quantitative values for the MUFA and PUFA contents (and SFA = 100 – PUFA – MUFA).

In addition to regions encompassing the olefinic and bis‐allylic bands, a further integral in the region 0.9–1.1 ppm is useful: this contains a resonance attributed to a double bond near the terminal CH_3_ in α‐linolenic acid (C18:3 ω‐3)^10^ that serves as a proxy for the total linolenic acid content. Although present only in small amounts in most vegetable oils (and only in trace amounts in argan oil^11^), linolenic acid's three unsaturated bonds make a contribution to the bis‐allylic and olefinic bands that needs to be adequately accounted for.

**FIGURE 2:**
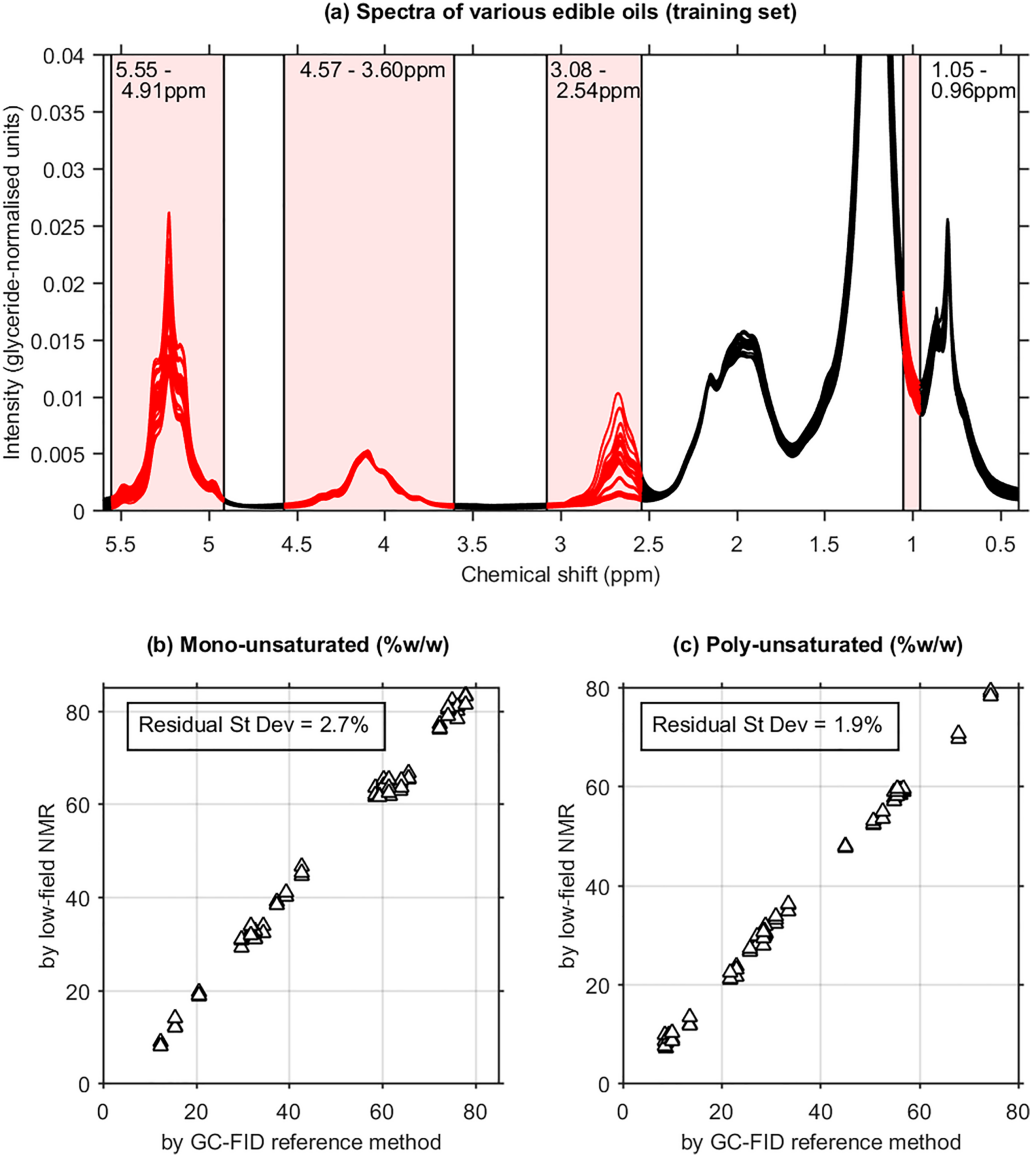
(a) 60‐MHz ^1^H nuclear magnetic resonance (NMR) spectra of a collection of edible oils used to develop calibrations for quantitative estimation of the fatty acid composition from selected integrated peak areas. The spectral regions used are indicated. (b) and (c) Results for respectively the monounsaturated and polyunsaturated contents obtained by applying the quantitation method to an independent test collection of edible oils. These samples were not used in developing the calibration equations. The reference composition values were obtained by gas chromatography–flame ionisation detector (GC‐FID; commercial contract placed with an independent accredited testing laboratory). The estimated error is ~2%w/w for both components

The V collection of assorted vegetable oils was used to establish calibrations for the MUFA and PUFA contents, by multiple linear regression onto the reference compositional values obtained by GC‐FID of FAMEs. The training set spectra are illustrated in Figure 2a, with the four regions used to calculate peak integrals as indicated. The calibration performances when applied to the independent test set are shown in Figures 2b and c.

**FIGURE 3:**
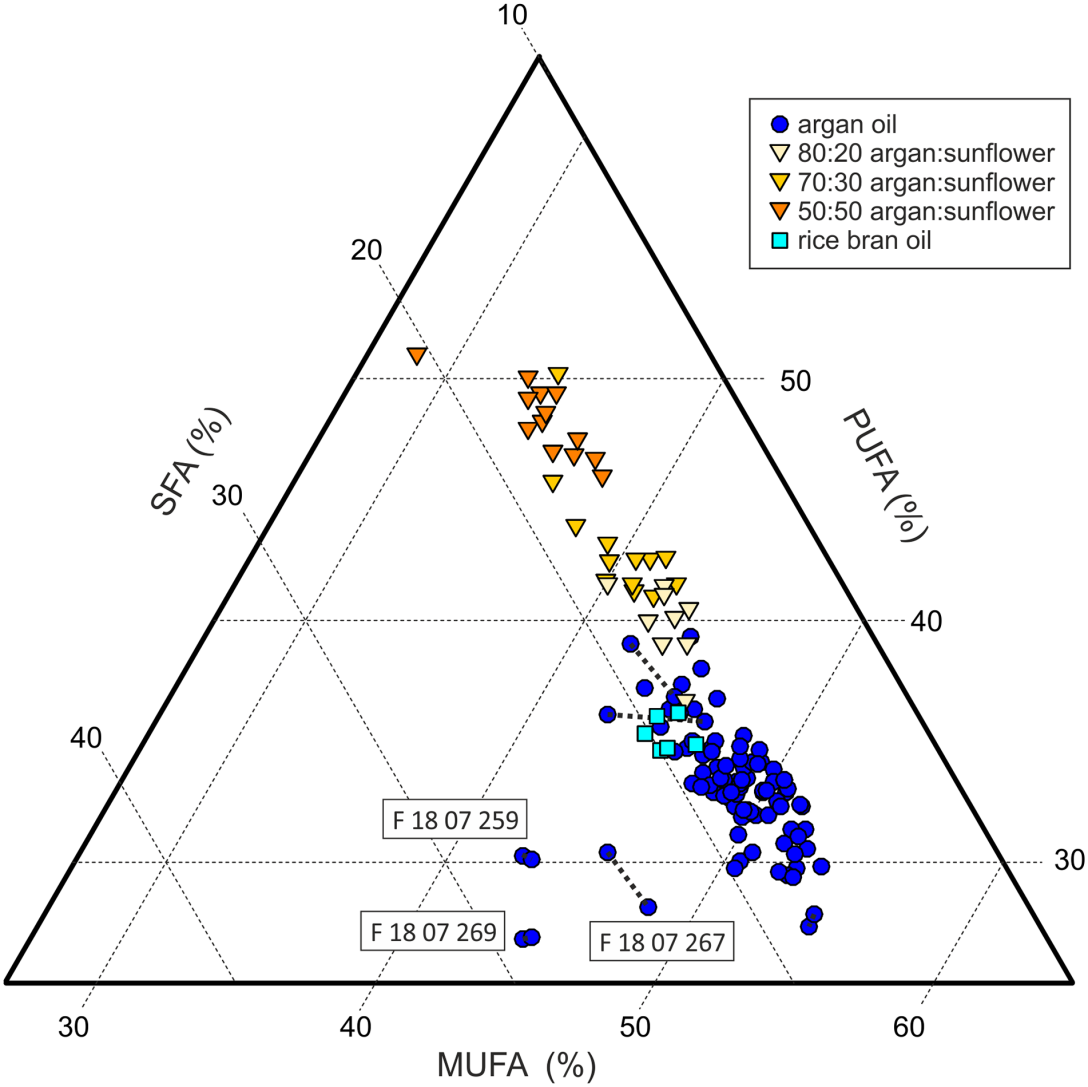
Monounsaturated fatty acid (MUFA), polyunsaturated fatty acid (PUFA) and saturated fatty acid (SFA) %w/w for collections ‘A’ and ‘Mnn’. The rice bran samples from collection ‘V’ are also depicted

The results of applying this quantitation method to the A collection of authentic argan oils are shown on the ternary plot in Figure 3 (blue filled circles), where MUFA, PUFA and SFA are expressed as a percentage of the TAG composition. As a natural product, variability in the proportions of MUFA/PUFA/SFA is expected, especially as our sample collection includes oils harvested at different times and from multiple growing regions. Kouidri, et al^12^ reported MUFA contents (as C16:1, C18:1 and C20:1) of 45.55 and 50.77%w/w, and PUFA contents (C18:2, C18:3) of 29.11 and 37.03%w/w, in oils from two different regions. As well as geographical origin, the fatty acid distribution is also known to vary with argan variety, year of harvest and harvest time.^13^ Aithammou, et al^14^ reported total UFA varying between 78.28 and 81.77%w/w (hence SFA between 18.23 and 21.72%w/w). The median MUFA content of our argan oils was 47%w/w [interquartile range (IQR) 3]; the PUFA content 32%w/w (IQR 3); and the SFA content 17%w/w (IQR 2). Thus, our results are in good agreement with these and other^15^ literature values.

Some of the duplicate measurements have been indicated, where clarity allows, by heavy dotted lines between points, to give an impression of the technical repeatability. The two main factors causing variance in peak heights/areas and the resultant compositional values are the shimming state of the spectrometer and the viscosity of the samples. Our standard operating protocol is to carry out a partial (‘XYZ’) shim at the start of each day's analyses. Line shape consistency could potentially be improved by more frequent shims but at the cost of a lengthier procedure overall.

Viscosity is a known cause of line broadening in NMR spectra, which most practitioners mitigate by dissolving viscous samples in a suitable solvent. We have previously used chloroform for this purpose in the analysis of olive and hazelnut oils.^5^ In contrast, the present work has been carried out using undiluted oils. The spectrometer magnet is maintained at a 37°C which gives a temperature inside the probe of ~35°C during acquisition. This reduces the viscosity of the sample compared with at room temperature and helps to ensure consistency across measurements.

Nevertheless, spectra from neat oils exhibit broader features than when diluted in for example chloroform. Crucially, however, we have found that this loss of resolution is of no consequence in terms of band integrals. Indeed, the repeatability across acquisitions was found to be slightly better in integrals calculated from neat oil spectra than in those diluted in solvent (Figure S1). Analysing the samples neat avoids expending time on a preparation step, as well as the costs and environmental concerns associated with solvents.

Three of the argan oils, as labelled on Figure 3, have clearly anomalous compositional values. Specifically, they have substantially higher proportions of SFA compared with the rest of the argan oils, although their MUFA/PUFA ratios (in the range 1.4–1.6) are comparable (1.2–1.8 for the A collection as a whole), and all are within the normal range for argan oils.^13^

**FIGURE 4:**
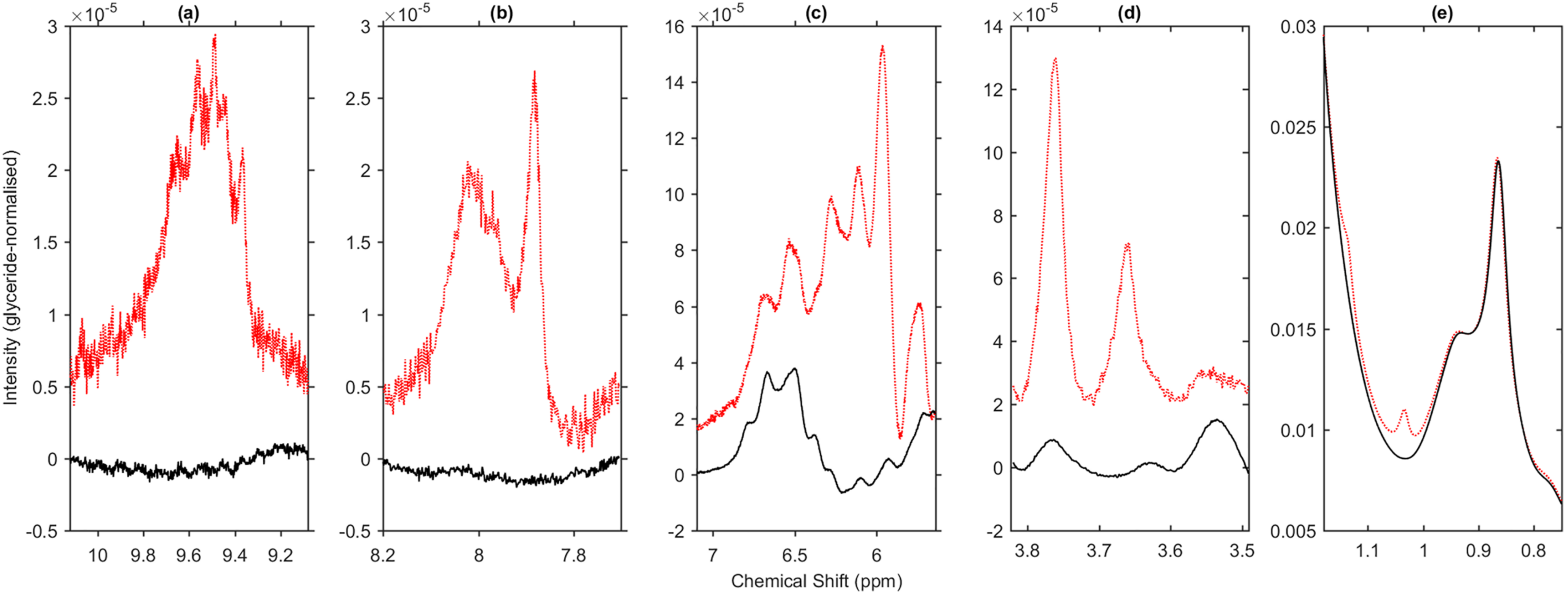
In panels (a)–(c), the red trace is the mean of spectra from the three oils with anomalous MUFA/PUFA/SFA profiles. In panels (d) and (e), the red trace is the mean of the duplicate spectra from a fourth sample with unusual peaks in the regions shown. In all panels, the black trace is the mean of the non‐anomalous argan oil spectra

Along with their unusual TAG profiles, these samples were also found to have additional small resonances in other regions of the spectrum. Figures 4a–c show three of these regions, comparing the mean of the three anomalous oils (red dotted line) with that of the remaining argan samples (black solid line). The spectra are again shown on a glyceride‐normalised scale: comparison with Figure 1 indicates that these features are orders of magnitude smaller than the olefinic or bis‐allylic bands. Some of the features between 6.3 and 6.9 ppm (Figure 4c) are found in all edible oil spectra and are likely due to minor compounds such as polyphenols^16^. However, the larger features between 6.0 and 6.5 ppm together with the peaks at 9.5–9.8 ppm are strongly suggestive of primary and secondary oxidation products.^17^ Notably, these three anomalous samples were amongst those that had been stored for several years before analysis—and whilst argan oil is relatively stable, degradation can occur and be accelerated by storage conditions^4^ or other factors such as time of harvest.^18^

Further additional peaks were found in a fourth sample with normal TAG composition, at ~1.03, 3.66 and 3.76 ppm (Figures 4d and e). We note that these correspond to characteristic peaks for beta‐sitosterol, stigmasterol and campesterol^16^, ^19^ and could be indicative of contamination at very low levels. However, definitive identification of such minor constituents is outside the scope of the present work.

Returning to Figure 3, also shown are the results of analysing the Mnn mixtures of 50:50, 70:30 and 80:20 argan with sunflower oil. The dependence of the TAG composition on the amount of adulterant is clear. This particular set of adulterated samples can be largely distinguished from authentic argan oils on the basis of their MUFA/PUFA/SFA profiles alone.

However, it would be misleading to extrapolate from this finding and suggest that TAG profiles provide a universal approach to detecting adulteration. First, the adulterated samples were prepared from a limited number of sunflower oils. Because the TAG composition of sunflower varies widely depending on cultivar and processing, many more examples would be needed to establish a robust detection limit. Second, certain other oil types are closer to argan oil in their TAG composition and would be much more challenging to detect by this approach. As an illustration, also included in the ternary plot are results from analysing the two rice bran oils (cyan filled squares, triplicate spectra) from the V collection. The TAG compositions of these oils are coextensive with those of normal argan oils, and these samples could not be identified as non‐argan by this method.

A screening method suitable for authentication requires a broader approach that also considers more subtle variances in the spectral profiles, for example, due to differences in fatty acid chain lengths and locations of unsaturated bonds, as well as signals from minor components. Whilst TAGs account for ~96% of an oil by weight, the remaining ~4% comprises low levels of compounds such as phenolics, tocopherols^20^ and free fatty acids.^21^ This type of information can be gleaned from the high‐field NMR spectrum,^22^ as discussed in the literature.^20^, ^21^, ^22^, ^23^ Because modern benchtop instruments are also high‐resolution instruments, the same information is present in the low‐field spectrum, but in overlapped form. Fortunately, the high‐quality data obtained are eminently amenable to processing using pattern recognition techniques, to extract and utilise the relevant information content.

The computational approach we have elected to use is based upon nearest‐neighbour outlier detection^24^ and is a variant of the method outlined in Antonides, et al..^25^ It is intuitively straightforward: items are deemed outliers if their nearest distances to members of the authentic class as measured by an ensemble of metrics exceed a collective critical value established for the class by empirical fitting.

Consideration needs to be given to the allocation of data when modelling the authentic class. We chose to establish the parameters of the class model using the ‘Run1’ spectra from 2/3 of the A samples (‘A‐train‐Run1’), selected by stratified sampling across the collection whilst excluding the four samples that exhibited anomalous additional peaks. Although the provenance of the argan oils is assured, the additional peaks suggest that their condition may be compromised, either by contamination during storage or degradation, given their age at the time of the NMR analysis. Thus, these samples were allocated to a test set (‘A‐test’) along with the remaining approximately one third of the authentic oils. The intended function of the class modelling is thereby a simultaneous authenticity and quality screen: samples found to be outliers are deemed compromised with respect to either or both.

**FIGURE 5:**
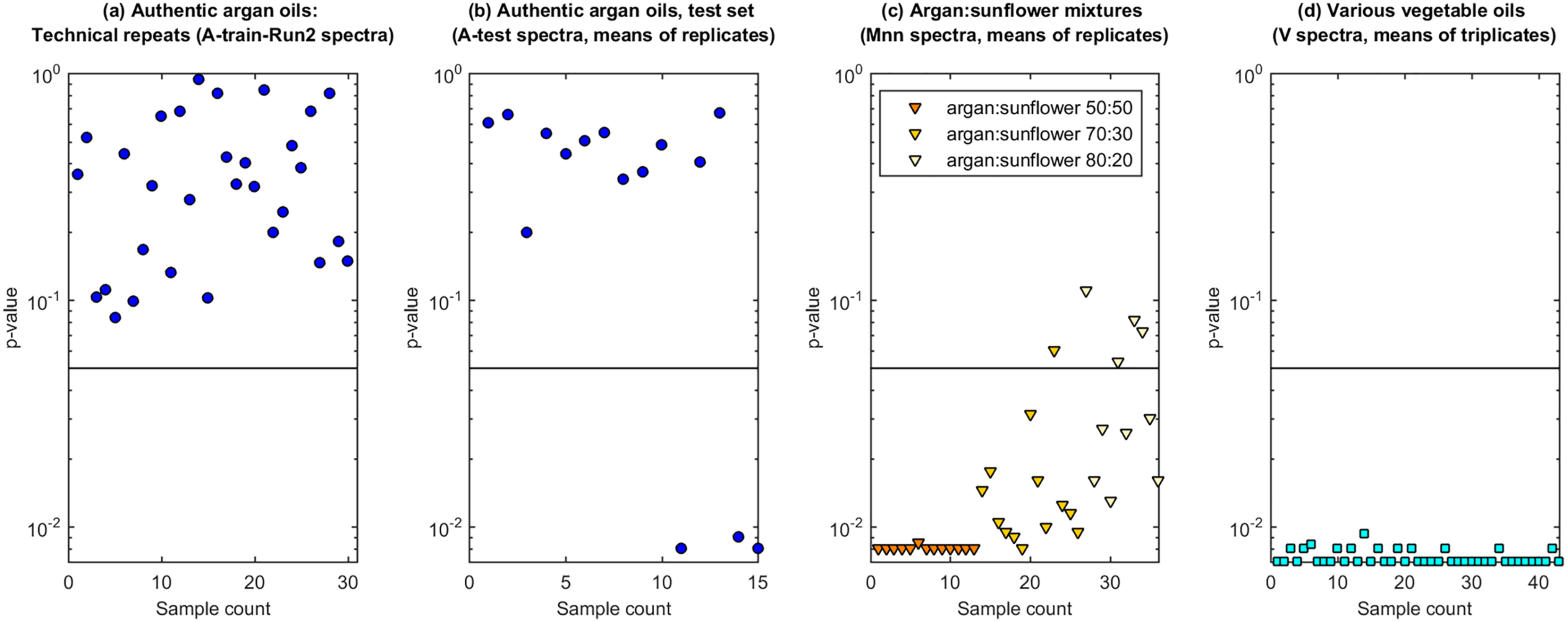
Results from applying the nearest‐neighbour class model to test samples. The accept/reject threshold is indicated by a horizontal line

The model was applied to various test sets, to gain an impression of its performance under different levels of challenge. The outcomes are presented graphically in Figure 5. The critical value for accept/reject was set at the *p* = .05 level. This is indicated on the figure, which uses a log *y*‐scale and expresses the outcome directly as the probability of incorrectly rejecting an authentic argan oil (Type 1 error).

The ‘A‐train‐Run2’ spectra are technical repeats of the A‐train‐Run1, and as anticipated, the model successfully identifies these as authentic argan (Figure 5a). For the remaining sample collections, the predictions were made from the means of replicate acquisitions, with outcomes as follows: of the A‐test samples, all but three were successfully accepted as argan (Figure 5b). The three outliers were the samples with unusual MUFA/PUFA/SFA profiles and additional anomalous peaks and thus a suspected quality issue. Note however that the fourth anomalous sample was accepted by the model.

Of the Mnn collections (Figure 5c), one of the M30 and four of the M20 collections were accepted as argan (Type 2 errors). This suggests a detection limit of around ~20%w/w sunflower in argan for these particular adulterated oils. The model was also challenged with the various vegetable oils used in the calibration work (V collection). All of these were correctly rejected (Figure 5d). Note that this includes the rice bran oils that had MUFA/PUFA/SFA profiles that overlapped with those of argan oils. Collectively, the outcomes from the class modelling suggest clear potential of benchtop ^1^H for screening argan oils for quality and authenticity.

**FIGURE 6:**
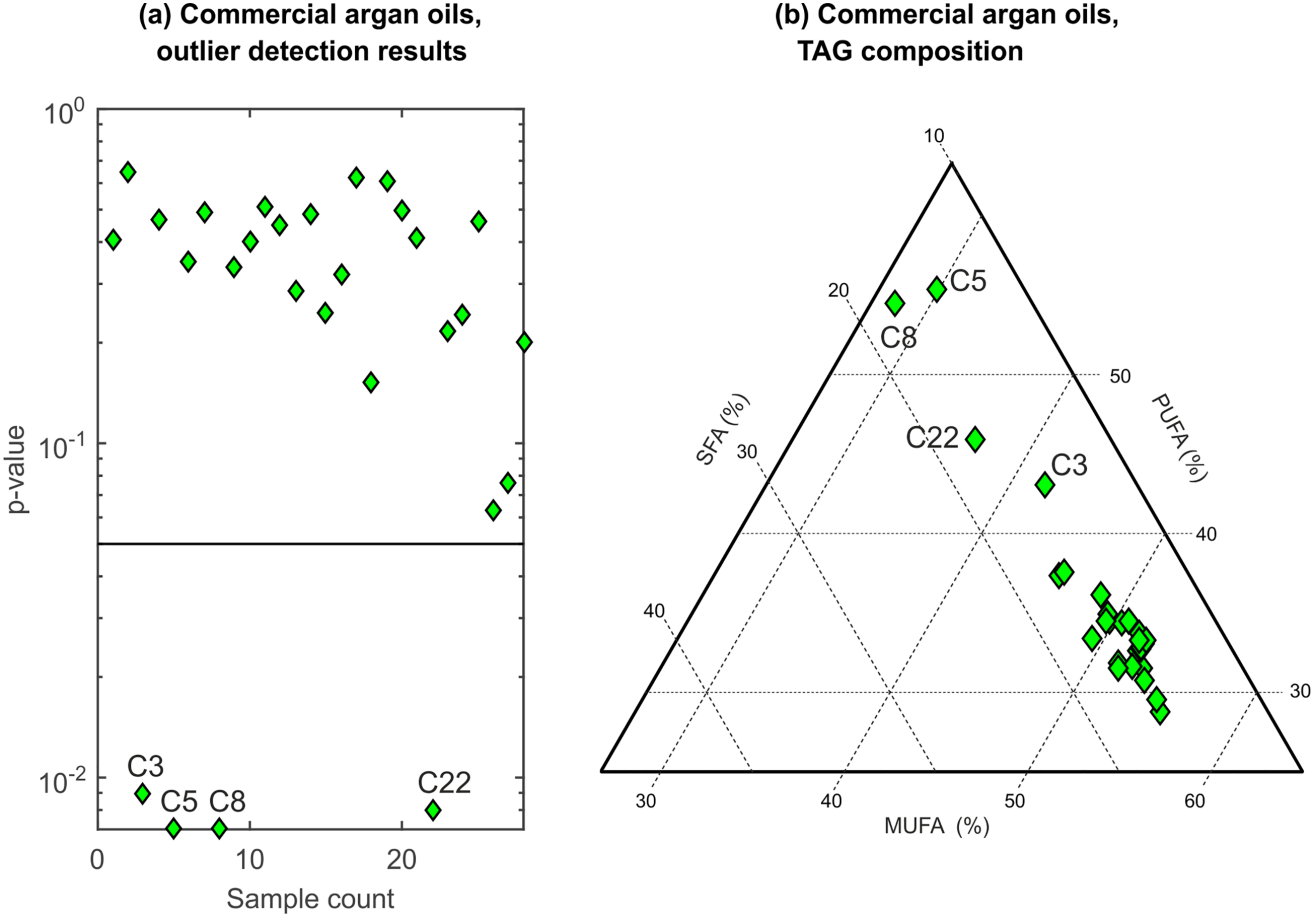
Results from applying (a) the nearest‐neighbour class model and (b) the MUFA/PUFA/SFA calibration, to the ‘C’ sample collection

Finally, the class model was applied to the collection of retail‐purchased argan oils. Of these, 24 were accepted as authentic; however, four were not (Figure 6a). Examination of their spectra showed clear differences in the spectral profiles, in all cases consistent with higher than expected levels of PUFAs; indeed, two of the samples showed evidence of significant α‐linolenic acid (see Figure S2) that is found only in trace quantities in authentic argan oil.^11^ The MUFA/PUFA/SFA profiles of these samples estimated by the quantitative TAG composition calibration were also found to be atypical for argan oils (Figure 6b). We conclude that these samples of 100% argan oil are adulterated, most likely with another vegetable oil.

## 4 CONCLUSIONS

Low‐field NMR spectroscopy of neat edible oils provides useful information about the sample composition. Selected peak integrals can be used to calibrate for the MUFA, PUFA (and SFA) composition. Applying this approach to a collection of authentic argan oils, we obtained typical values for the MUFA and PUFA contents of 47 and 32%w/w. This analytical test is fast (5 mins per sample), requires no sample preparation or solvents and gives information with an accuracy and precision suitable for ‘typical value’ food labelling.

With regards to authentication, the MUFA/PUFA/SFA approach provides a simple method of examining for compositional anomalies, but it does not make use of the whole spectrum and is thus unable to detect more subtle differences between oil types or the presence of contaminants or other signals that could indicate a quality issue. An outlier detection approach was employed that makes use of information from across the whole spectral range. This was able to successfully accept good quality, authentic argan oils as such, whilst rejecting all but a small proportion of the ‘test’ adulterated samples, and comprehensively rejecting a further 43 vegetable oils of 15 different types.

A survey was conducted of 28 retail‐purchased oils labelled as 100% argan. Using the class model, four of these were identified as outliers. Further investigation showed that this was due to very clear differences in their TAG composition in comparison to the normal MUFA/PUFA/SFA profile for argan oils. Two of these showed clear evidence of elevated amounts of α‐linolenic acid, which is largely absent from argan oil. These findings are consistent with the oils being adulterated with other vegetable oils, at levels suggestive of fraud for economic gain. By standard sampling theory, this rate of fraud (4 out of 28 samples) corresponds to an estimated percentage for the sector of 14% [confidence interval (CI) 7–23%].

## Supporting information

Data S1 Supporting information
