# Supplementary material for: High‐throughput screening of argan oil composition and authenticity using benchtop 1H NMR
Source: Magn Reson Chem. 2020 Apr 6;58(12):1177–86. doi: 10.1002/mrc.5023 (PMC8653893; doi:10.1002/mrc.5023)
Supplement: Supplementary file 1 — Data S1 Supporting information [file MRC-58-1177-s001.doc]

SUPPORTING INFORMATION - Pseudocode for outlier detection method.

**algorithm:** generate one-class model

**inputs:** *xall* - [*n* x *p*] matrix of data row vectors that are true members of the class (i.e. each row contains one spectrum; in our implementation, *xall* is variance-scaled; the scaling parameters are stored for use with future test data).

*distancemetrics* – list of *d* different metric types to use (in our implementation, *d* = 3 and the metrics are ‘Euclidean’, ‘correlation’ and ’Spearmans’ distances)

**outputs:** *distecdfs, classcdf –* [*g* x *d*] matrix and [*g* x 1] vector of empirically fitted cumulative distribution functions used for testing nearest neighbour distances calculated from new input data

**for** *i*th string in *distancemetrics* (*i* = 1…*d*)

**for** *j*th row in *xall (j = 1…n)*

**for** *k*th row in *xall (k = 1…n)*

Calculate the distance between *j*th and *k*th rows of xall as measured using *i*th distance metric*.*

Store the distance as (*j*th, *k*th) element in [*n* x *n*] matrix *alldists*

**end for**

**end for**

**for** *j*th row in *alldists (j = 1…n)*

Extract the distance of the *j*th cross-validation item from its nearest neighbour: *outernndist(j)* = minimum value in *j*th row of *alldists (*excluding trivial element (*j*,*j*) which is equal to zero)

Extract all the (non-trivial) nearest neighbour distances of the not(*j)* items: *innernndists* = minimum values in each of the not(*j)* rows in *alldists*

Fit and store an empirical cumulative distribution function at desired gridding to the values in *innernndists*

Use the ecdf to get a percentile value for *outernndist(j)* and store as (*j*th, ith) element in an [*n* x *d*] matrix *percvals*

**end for**

Store the median of the ecdfs for the current distance metric in a [*g* x *d*] matrix *distecdfs*, where *g* is the number of grid points

**end for**

Form a statistic by combining the *d* columns of *percvals* into a single [*n* x 1] vector, such as *meanstat* = mean(*percvals^T^*)

Use a Gaussian copula with correlation parameters as for *percvals* to generate a large sample of random numbers from which to empirically determine the cumulative distribution function of *meanstat*. Store this as a [*g* x 1] vector *classcdf. A*long with *distecdfs*, this is used to test the nearest neighbour distances of new items from the class, directly returning a p-value for H0 = item is a member of the class

**return**

SUPPORTING INFORMATION - Figure 1.


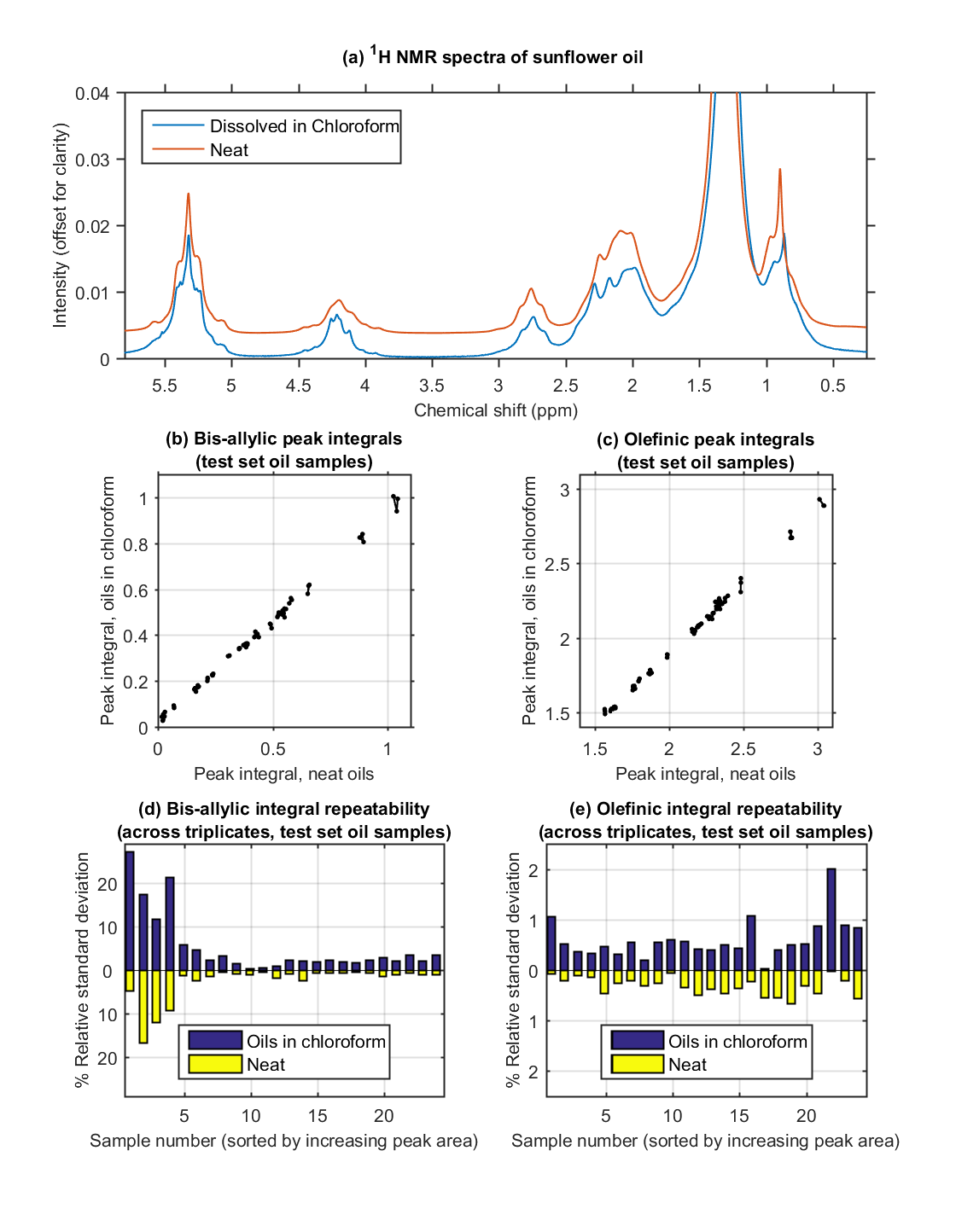


(a) 60 MHz 1H NMR spectra of sunflower oil, collected neat and dissolved in chloroform.

(b) and (c): comparison of respectively the bis-allylic and olefinic region integrals, calculated for the ‘V-test’ collection of edible oils used in developing the quantitative MUFA/PUFA/SFA calibrations. Convex hulls are drawn around the triplicates for each sample. In almost all cases, the extent of these is greater along the y-axis than the x-axis, indicating that the measurement repeatability is better for neat oils than for diluted.

This is confirmed by figures (d) and (e), which show, for respectively the bis-allylic and olefinic regions, the % relative standard deviation for the triplicate repeats of each sample, calculated separately for the dilute and neat spectra and compared directly by the bar plots. For almost all samples, the %RSD is lower when the samples were analysed neat.

SUPPORTING INFORMATION - Figure 2.


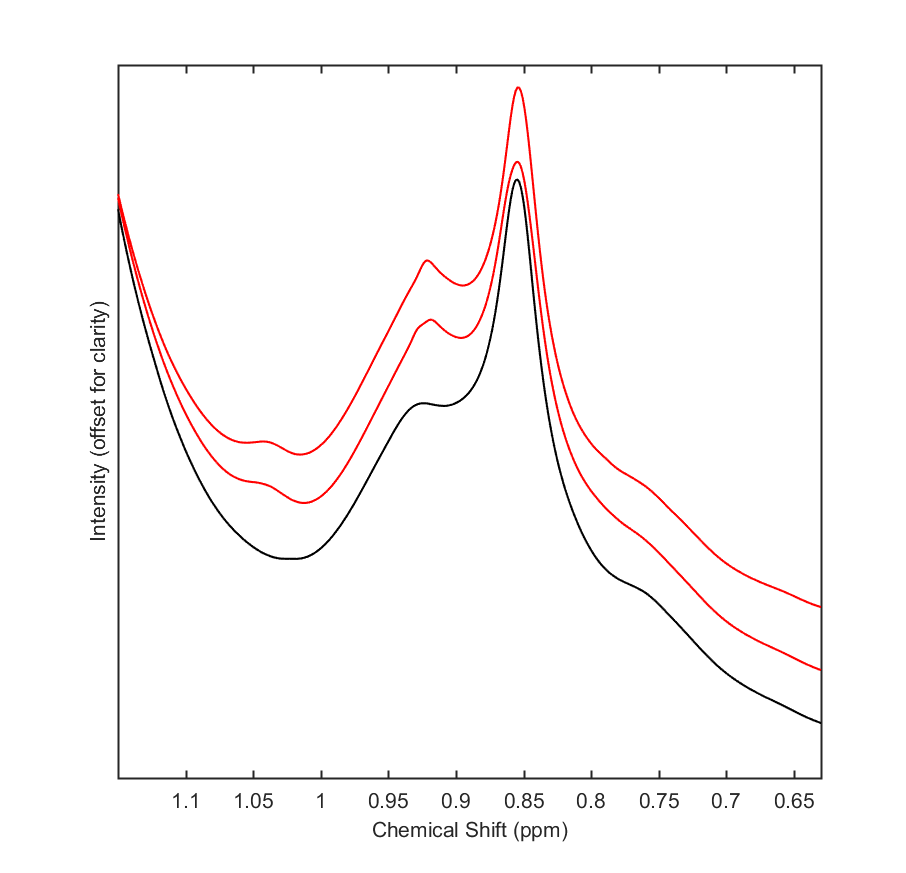


Spectra of a typical argan oil (black trace) and two of the four ‘outlier’ commercial oils (red traces, samples C05 and C08). The feature at ~1.05 ppm is one of a triplet of resonances attributable to α-linolenic acid, as is the difference in band shape at ~0.9ppm (see Jakes et al, Food Chem. 2015, 175, 1-9).
